# Bioinformatics analyses of potential ACLF biological mechanisms and identification of immune-related hub genes and vital miRNAs

**DOI:** 10.1038/s41598-022-18396-1

**Published:** 2022-08-18

**Authors:** Jiajun Liang, Xiaoyi Wei, Weixin Hou, Hanjing Wang, Qiuyun Zhang, Yanbin Gao, Yuqiong Du

**Affiliations:** 1 School of Traditional Chinese Medicine, Capital Medical University, Beijing, China; 2 Beijing Key Laboratory of TCM Collateral Disease Theory Research, Beijing, 100069 China

**Keywords:** Genome informatics, Hepatitis, Liver diseases, Hepatology

## Abstract

Acute-on-chronic liver failure (ACLF) is a critical and refractory disease and a hepatic disorder accompanied by immune dysfunction. Thus, it is essential to explore key immune-related genes of ACLF and investigate its mechanisms. We used two public datasets (GSE142255 and GSE168048) to perform various bioinformatics analyses, including WGCNA, CIBERSORT, and GSEA. We also constructed an ACLF immune-related protein–protein interaction (PPI) network to obtain hub differentially expressed genes (DEGs) and predict corresponding miRNAs. Finally, an ACLF rat model was established to verify the results. A total of 388 DEGs were identified in ACLF, including 162 upregulated and 226 downregulated genes. The enrichment analyses revealed that these DEGs were mainly involved in inflammatory-immune responses and biosynthetic metabolic pathways. Twenty-eight gene modules were obtained using WGCNA and the coral1 and darkseagreen4 modules were highly correlated with M1 macrophage polarization. As a result, 10 hub genes and 2 miRNAs were identified to be significantly altered in ACLF. The bioinformatics analyses of the two datasets presented valuable insights into the pathogenesis and screening of hub genes of ACLF. These results might contribute to a better understanding of the potential molecular mechanisms of ACLF. Finally, further studies are required to validate our current findings.

## Introduction

Acute-on-chronic liver failure (ACLF) is a major form of liver failure and a chronic liver disease syndrome with acute decompositions that presents a rapid progression and high mortality. The clinical symptoms of ACLF patients include jaundice, ascites, hepatic encephalopathy, or extrahepatic manifestations such as coagulopathy, acute kidney injury, or sepsis^1^. A meta-analysis that covered 30 cohort studies worldwide showed that the 28- and 90-day mortality rates of ACLF patients were 45 and 58%, respectively^2^. However, besides liver transplantation, there is no definitive and effective treatment for ACLF. Moreover, the specific treatment for this life-threatening disease has been restricted by the lack of knowledge regarding its molecular mechanisms.

Recently, various bioinformatics approaches have been used to explore the molecular mechanisms of ACLF. Through bioinformatics, massive samples can be processed in a short time to uncover underlying biological mechanisms^3^. Previous studies have used transcriptome analysis of PMBS, neutrophils, and liver tissues to screen and identify differential genes and obtain characteristic mRNA and miRNA expression profiles for ACLF. They found that ELANE, MPO, and CD177 were upregulated and proposed a miR-6840-3p-JADE2 mRNA-miRNA interaction network that affects the prognosis of ACLF patients^4,5^. Additionally, a previous genomic study has identified abnormal expression profiles of neutrophils, macrophages, and lymphocytes^6^. These extensive gene expression reports are of great significance for the study of ACLF. However, specific gene modules with certain biological functions of ACLF have not yet been analyzed, which is equally important for potential ACLF treatment strategies.

**Figure 1:**
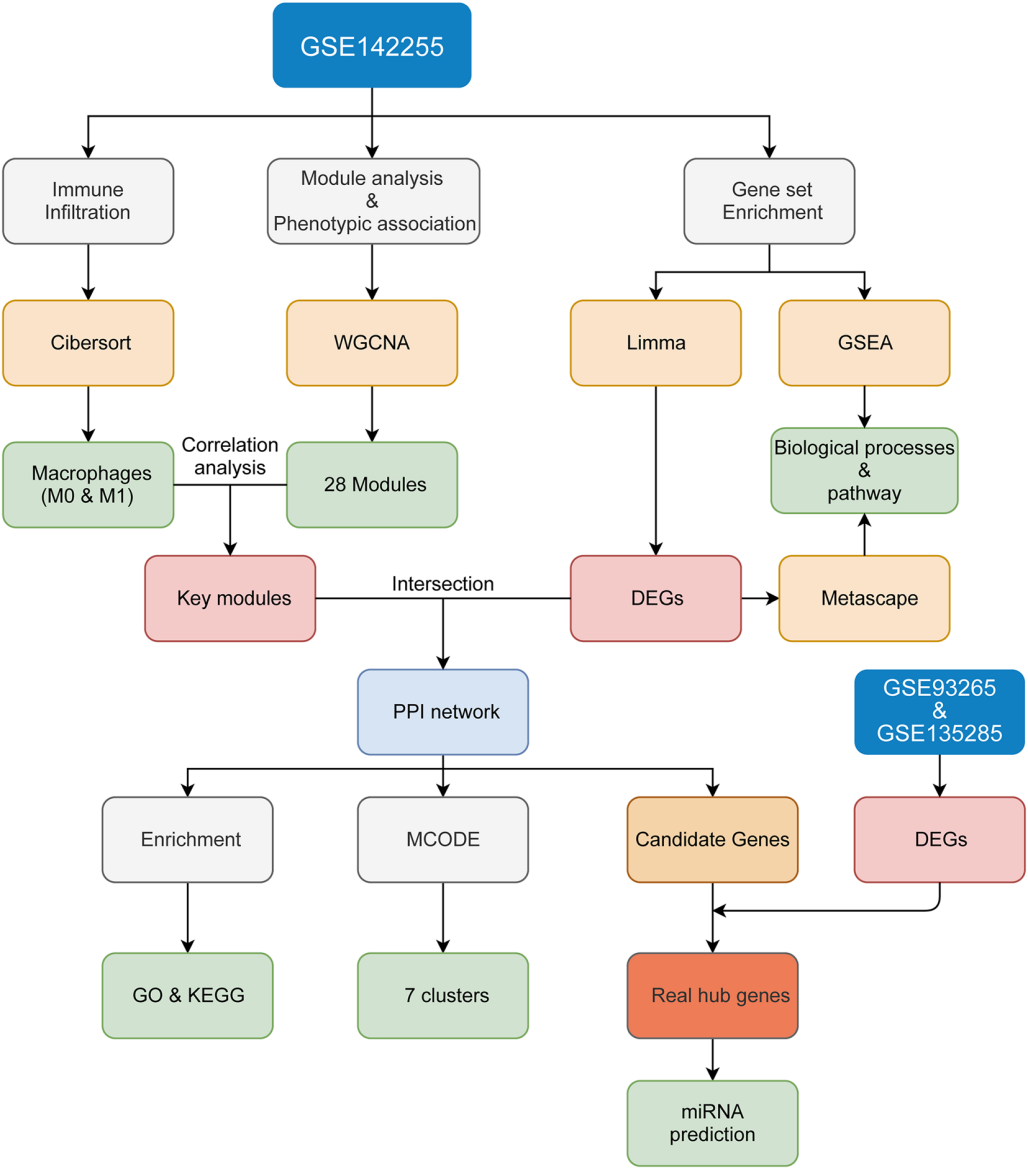
Flow chart of bioinformatics analyses for ACLF.

Weighted correlation network analysis (WGCNA) can be used to find co-expressed gene modules and explore the correlation between gene networks and disease phenotypes by assuming that gene networks follow a scale-free distribution and clustering genes with similar expression patterns^7^. The CIBERSORT algorithm can be used to analyze immune cell composition from complicated tissue gene expression profiles to estimate immune infiltration based on RNA-seq data^8^. This algorithm applies to gene expression profiles of almost all tissues and covers a wide range of immune cell types, being widely used for estimating the abundance of immune cells in various tissues. In the present study, we used multiple bioinformatics approaches to analyze ACLF-related microarray datasets in the GEO database, identify key immune-related genes, and provide novel strategies for ACLF treatment. We identified the immune cells involved in ACLF, constructed gene co-expression modules associated with these immune cells and hub genes, and predicted reciprocal miRNAs. Finally, the expression of these hub genes and miRNAs was analyzed using an ACLF rat model. Our current findings might provide a valuable reference for the pathogenesis and potential molecular targets of ACLF, and can contribute to the development of therapeutic targets and corresponding drugs for this disease. The workflow of this study is shown in Fig. 1.

## Materials and methods

### Acquisition and processing of the gene expression matrix

Here, we retrieved clinical samples of ACLF from the GEO database (http://www.ncbi.nlm.nih.gov/geo), a public functional genome database that preserves gene expression and microarray data (Table 1). Twenty-four whole blood samples were selected from GSE142255, comprehending 7 healthy subjects (HS) and 17 ACLF patients. Besides, the GSE168048 dataset was used for validation and described the 28-day survival status of HBV-ACLF patients (8 surviving cases and 8 dead controls). All samples were from *Homo sapiens*.

**Table 1:** Summary of the three ACLF datasets from the GEO platform.

GEO gene set ID	GSE142255	GSE168048
Title	Gene expression profiling of immune cells from patients with acute on chronic liver failure (ACLF)	Prognosis associated mRNA in peripheral blood mononuclear cells (PBMCs) from hepatitis B virus-related acute-on-chronic liver failure (HBV-ACLF)
Platform	GPL17586: [HTA-2_0] Affymetrix Human Transcriptome Array 2.0 [transcript (gene) version	GPL21185: Agilent-072363 SurePrint G3 Human GE v3 8 × 60 K Microarray 039,494 [Probe Name Version]
Samples	7 healthy subjects and 17 ACLF patients	8 survival cases and 9 dead controls
Sample type	Whole blood	Whole blood

### Differentially expressed genes (DEGs)

The identification of DEGs was performed using the “limma” R package provided by Sangerbox tools, a free online platform for data analyses (http://www.sangerbox.com/tool). The *p*-values and adjusted *p*-values were calculated using t-tests. Genes in each sample that met |log_2_ (fold change) |> 1 and adjusted *p* < 0.05 were retained.

#### Enrichment analyses

Gene Set Enrichment Analysis (GSEA) is a computational method used to determine whether a pre-defined gene set can exhibit consistent significant differences in two biological states^9^. The c2.cp.kegg.v7.4.symbols.gmt was chosen as a control to assess ACLF-related pathways. Based on gene expression profiles and phenotypic groupings, a minimum gene set of 5 and a maximum gene set of 5000 was selected. One thousand resamplings were performed and a *p-*value < 0.05 and an FDR < 0.25 were considered statistically significant. Then, to explore which pathways were involved in ACLF pathogenesis, the complete gene expression matrix information of GSE142255 was uploaded to GSEA, and the enrichment results satisfying |NES|≥ 1.0 and NOM *p* < 0.05 were considered statistically significant. The Metascape^10^ database and KEGG database (https://www.kegg.jp/kegg/kegg1.html) were used to perform Gene Ontology (GO) and KEGG pathway enrichment analyses by submitting specific gene lists, including upregulated and downregulated DEGs, with min overlap = 3, min enrichment = 1.5, and *p* < 0.01. Besides, biological processes (BP) networks of DEGs were analyzed and visualized using Cluego in Cytoscape^11^. The kappa score was set as 0.4. GO terms with at least 9 gene hits and gene percentages > 9% were selected and a *p* < 0.05 was considered statistically significant.

### Immune cell infiltration analysis and WGCNA

CIBERSORT is an algorithm for estimating cellular compositions in tissues based on normalized gene expression data^12^. In the current study, whole blood mRNA expression profile data from ACLF patients and HS were extracted from GSE142255. CIBERSORT was used to assess the relative proportions of 22 immune cells. The CIBERSORT method was chosen to calculate the immuno-infiltrating cell scores for each sample based on the expression profiles obtained using the IOBR R package. To identify gene co-expression modules, the top 15,000 genes with the most significant differences between ACLF patients and HS were used to construct a co-expression network via the WGCNA package provided by Sangerbox tools^13^. The proper power was calculated based on the pickSoftThreshold function. Gene profiles were considered as unsigned co-expression networks. The execution parameters were: power = 14, blockSize = 7000, minModuleSize = 20, deepSplit = 2, mergeCutHeight = 0.25, hub cut = 0.9, net threshold = 0. The gene co-expression modules obtained were further used for association analysis with primarily infiltrating immune cells indicated by CIBERSORT. The correlations between modules and immune infiltrative features were analyzed and presented as heat maps. The grey module represents a collection of genes that could not be assigned to any module. Gene Significance (GS) represents the association between gene expression and each feature, while Module Membership (MM) is the correlation of gene expression with each module. The correlation between MM and GS was calculated using Pearson correlation analysis. The identification of gene clusters and the protein–protein interaction (PPI) network analysis were also performed. Intersecting genes were obtained from the most relevant modular DEGs and uploaded to Metascape for PPI network construction, and GO and KEGG pathway enrichment analyses. A *p* < 0.05 was considered statistically significant.

### Screening of hub genes and validation with the GSE168048 dataset

CytoHubba is a Cytoscape plugin used to analyze and acquire key genes in a network and provides various topology analysis algorithms, including Degree, Edge Percolated Component (EPC), Maximum Neighborhood Component (MNC), Density of Maximum Neighborhood Component (DMNC), Maximal Clique Centrality (MCC), Bottleneck (BN), EcCentricity, Closeness, Radiality and Betweenness^14^. In the present study, each node was ranked using these ten topological analysis algorithms. Using the "UpSet" R package, hub genes were filtered according to the first 60 genes in each algorithm and verified using the GSE168048 dataset.

### Construction of a potential miRNA-target regulatory network

We used miRnet 2.0 (https://www.mirnet.ca/) to search for the corresponding miRNAs of hub genes and visualize the miRNA-target regulatory network^15^. The miRNAs with 2 or more degrees were displayed.

### Animals and ACLF model

A total of 18 Wistar rats (180–220 g) were purchased from Beijing Vital River Laboratory Animal Technology Co., Ltd., Beijing, China. After one week of acclimatization, rats were randomly divided into two groups: normal control (NC, n = 9) and ACLF (ACLF, n = 9). The ACLF rat model was established as previously described. Briefly, rats were intraperitoneally injected with 40% carbon tetrachloride (CCl_4_) olive oil solution (1.5 ml/kg) twice a week for ten weeks and an acute liver injury was induced by intraperitoneal injection of LPS (100 μg/kg) and D-GalN (400 mg/kg). After 12 h, all rats were sacrificed and the livers were collected and stored at − 80 ℃. Histological observation.

To make tissue sections, rat liver tissues were first embedded in paraformaldehyde, then in paraffin. Processed tissue sections were stained with hematoxylin–eosin (HE) and Masson.

### Immunofluorescence staining

Sections were dewaxed with xylene and ethanol, and antigen-repaired with citric acid buffer. Tissues were blocked with 10% goat serum for 1 h and co-incubated with iba1 (1:100, sc-32725, Santa Cruz, USA) and CD68 (1:100, ab283654, Abcam, UK) antibodies at 4 °C. After rinsing with PBST, the sections were incubated with CoraLite488-conjugated Goat Anti-Mouse IgG(H + L) (1:500, SA00013-4, Proteintech, USA) and CoraLite594—conjugated Goat Anti-Rabbit IgG(H + L) (1:500, SA00013-4, Proteintech, USA) for 2 h at 37 °C. After rinsing, tissue sections were covered with an antifade mounting medium with DAPI and observed using a Leica TCS SP8 STED 3X Super-Resolution Confocal Microscope (Leica, Germany). Ten fields of view were randomly selected for each liver section (× 400).

### Quantitative real-time PCR (qRT-PCR) analysis

Briefly, the RNA was extracted from livers using Trizol and 50 µL of RNase-free water was added to lyse the RNA. The RNA was reverse transcribed to cDNA by adding primers synthesized by the Beijing Tianyi Huiyuan Biotechnology Co., LTD. Then, the qRT-PCR reaction was performed using the TB Green® Premix Ex Taq™ II (Tli RNaseH Plus) kit mixed with cDNA. The reaction conditions comprehended 40 cycles of 95 °C for 10 s, and 60 °C for 30 s after pre-denaturation for 5 min. The 2^−ΔΔCt^ method was used to analyze the expression of mRNAs and miRNAs. The mRNA expression was normalized to β -actin, while miRNA was normalized to U6. The primer sequences are shown in Table 2.

**Table 2:** The sequences of primers.

Gene name	Primer Sequences (5’ to 3’)
β-actin	Forward: GAAGTGTGACGTTGACATCCG
	Reverse: GCCTAGAAGCATTTGCGGTG
RSL1D1	Forward: TGATGAACGAATCCGACGGCATT
	Reverse: CTCCATTCCAGTGTGACCAATACG
RPS5	Forward: AAGACCATCGCTGAGTGCCTTG
	Reverse: GCCACACGCTCCAGTTCATCT
CCL5	Forward: CTCGAAGGAACCGCCAAGTGT
	Reverse: GGACTAGAGCAAGCAATGACAGGA
HSPA8	Forward: ACCAGACTGCGGAGAAGGAAGAAT
	Reverse: AGAAGCACCACCAGATGGAGGAG
PRKCQ	Forward: AGCCTCCTGAACCTGAAGTGAACT
	Reverse: CGTCATCGTCCATCAACACCACAT
MMP9	Forward: GCCTACGTGACCTATGACCTCCT
	Reverse: GCCTCCACTCCTTCCTAGTCTCTA
ITGAM	Forward: GTGTCAGCAAGCCAGAACCAGTT
	Reverse: CACAACGACCTTGAGGAGCAGTT
LCK	Forward: CGCCATTACACCAACGCCTCTG
	Reverse: GCCGCTCAACCAACTTCAATGTCT
IL7R	Forward: AGGATGTCAGTGGTGGGTCTATCA
	Reverse: CAAGGAGGGTTGAAGTTGGAATGC
HP	Forward: GTGAGAATGCGACAGCCAAGGA
	Reverse: AGGCAGGCAGATAGGCATGACT
U6	Forward: CTCGCTTCGGCAGCACA
	Reverse: CGCTTCACGAATTTGCGT
mir-9a-5p	Forward: GCCGAGTCTTTGGTTATCTAGCT
	Reverse: GTGTCGTGGAGTCGGCAATTC
miR-16-5p	Forward: GCCGAGTAGCAGCACGTAAATA
	Reverse: GTGTCGTGGAGTCGGCAATTC
miR-182	Forward: GCCTTTGGCAATGGTAGAACTC
	Reverse: GTGTCGTGGAGTCGGCAATTC
miR-26a-5p	Forward: GCCGAGTTCAAGTAATCCAGGA
	Reverse: GTGTCGTGGAGTCGGCAATTC

### Ethical approval

All animal experiments were performed according to ARRIVE guidelines and carried out in the Experimental Animal Center of Capital Medical University. All protocols were carried out in accordance with current legislation relating to animals and experiments involving animals and were approved by the Animal Experiments and Experimental Animal Welfare Committee of Capital Medical University (Beijing, China).

## Results

### Screening of DEGs

**Figure 2:**
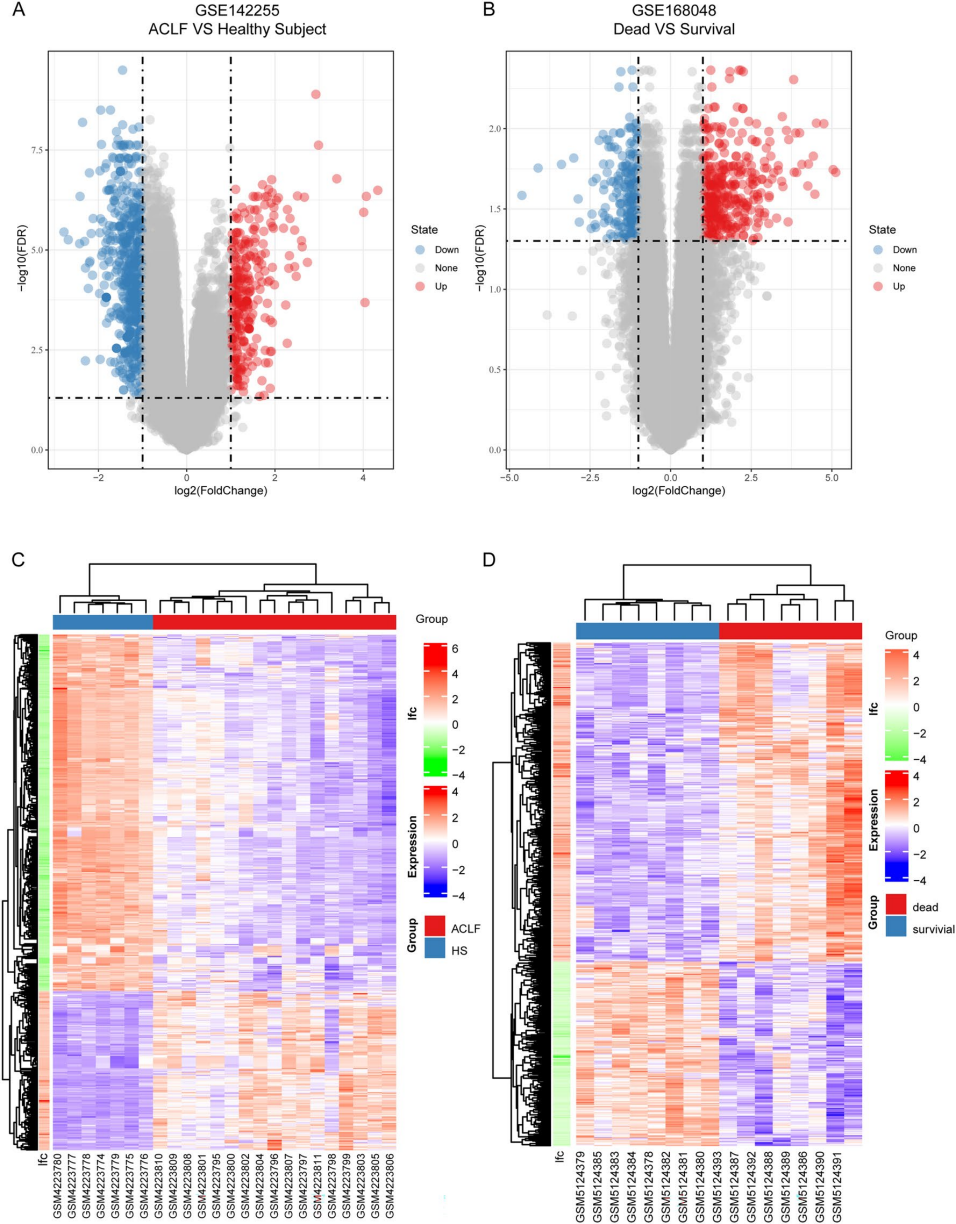
Visualization of gene expression on GSE142255 and GSE168048. Differentially expressed genes (DEGs) in GSE142255 (**A**) and GSE168048 (**B**) were screened with |log2 (fold change) |> 1 and adjusted *p* < 0.05 and are presented in a Volcano plot. Blue plots represent downregulated DEGs and red plots represent upregulated ones. The expression of DEGs of each sample in GSE142255 (**C**) and GSE168048 (**D**) was visualized in clustered heat maps.

Two microarray datasets (GSE12255 and GSE168048) were used to screen DEGs with the following criteria: adjusted *p*-value < 0.05 and |log_2_ FC|> 1. A total of 388 DEGs (226 downregulated and 162 upregulated) were identified after removing unannotated probes and duplicated genes in GSE142255 (Fig. 2A,C, and Supplementary Table S1). Meanwhile, 386 DEGs were upregulated and 223 were downregulated in the GSE168048 dataset (Fig. 2B,D, and Supplementary Table S2).

### Pathway enrichment analysis and functional annotation of GSE142255

**Figure 3:**
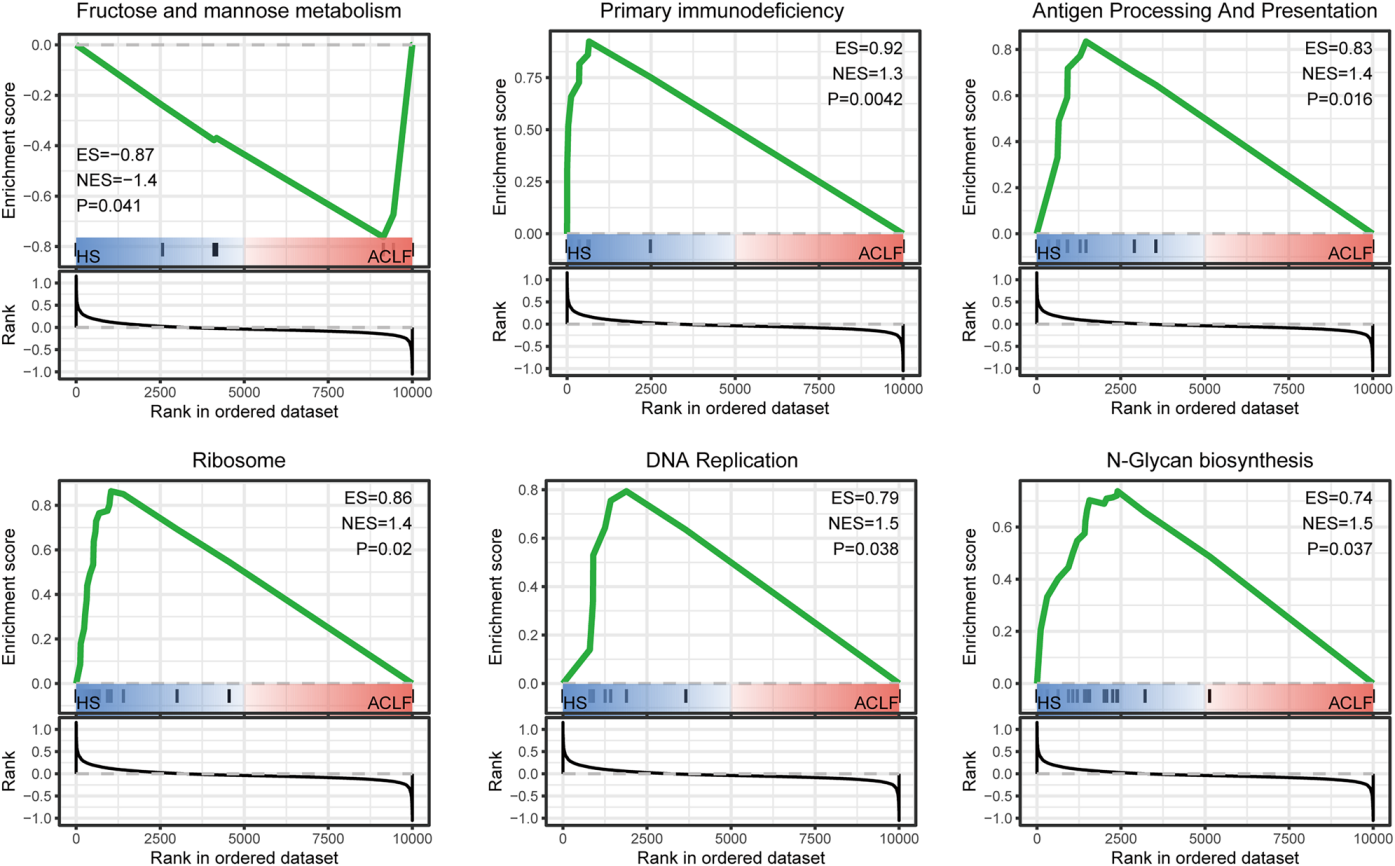
Six significantly enriched gene sets of ACLF patients in GSE142255 through Gene Set Enrichment Analysis (GSEA).

**Figure 4:**
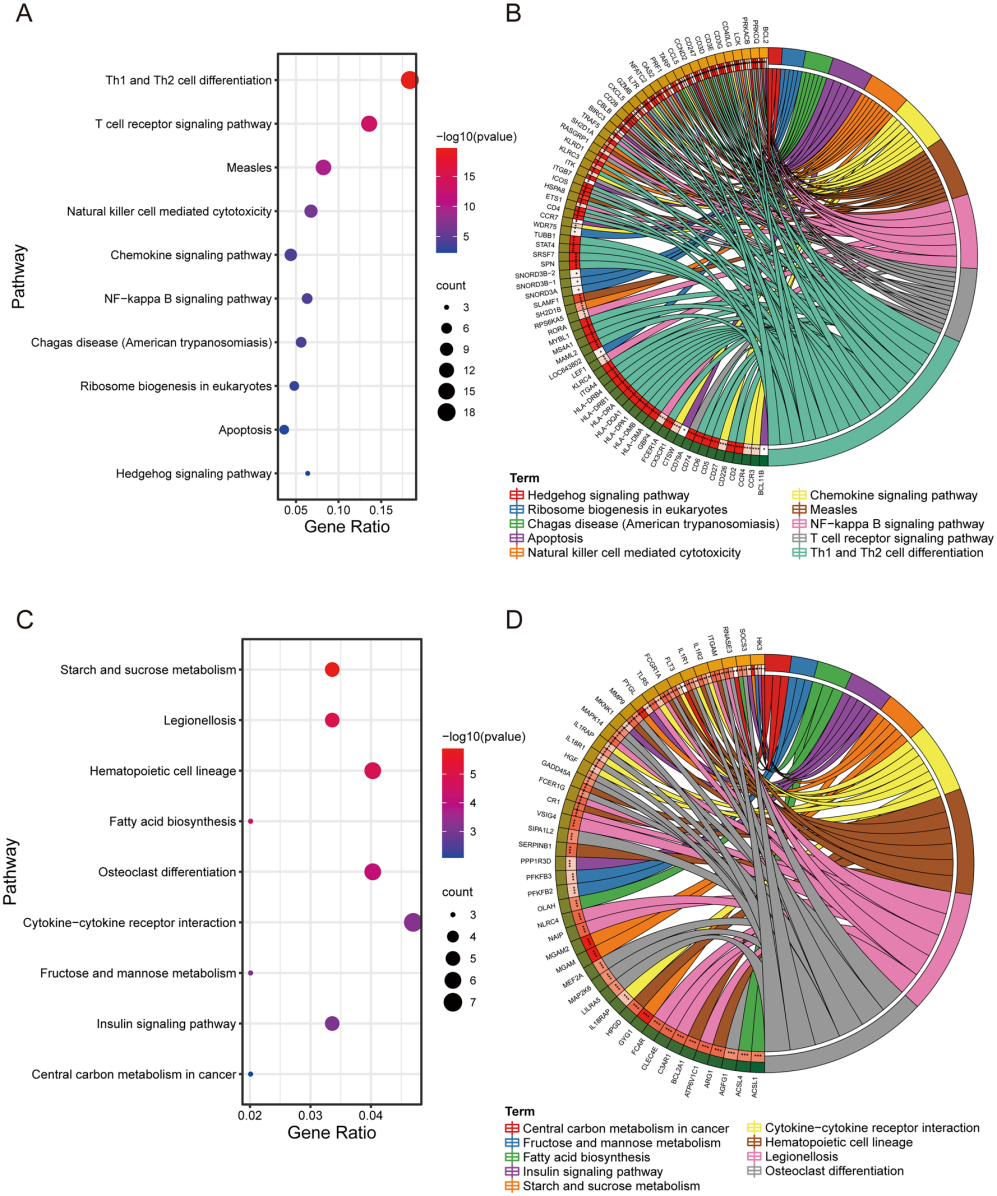
KEGG enrichment analysis of GSE142255 DEGs. The top 10 enriched pathways of downregulated (**A**) and upregulated (**C**) DEGs were visualized in a bubble map. The relationship between downregulated (**B**) or upregulated (**D**) DEGs and pathways were visualized in a string diagram.

All annotated gene information of ACLF and HS in GSE142255 was uploaded to the GSEA software for analysis at a holistic level. Pathways with |NES|≥ 1.0 and NOM *p*-value < 0.05 were considered as significantly enriched gene sets. The GSEA results revealed significant enrichment in immune-related functions and biosynthesis metabolic pathways (Fig. 3), including primary immunodeficiency, and fructose and mannose metabolism. Using Metascape, 162 upregulated and 226 downregulated DEGs were enriched in GSE142255 according to the KEGG pathway analysis (Supplementary Table S3). Downregulated DEGs were mainly engaged in multiple immune responses and inflammatory signaling pathways, including Th1 and Th2 cell differentiation, T cell receptor signaling pathway, Natural killer cell-mediated cytotoxicity, Chemokine signaling pathway, NF-kappa B signaling pathway, and Apoptosis. Meanwhile, upregulated DEGs affected biosynthetic and substance metabolism pathways, including Starch and sucrose metabolism, MAPK signaling pathway, Fatty acid biosynthesis, Insulin signaling, Cytokine-cytokine receptor interaction, Fructose and mannose metabolism, and Central carbon metabolism in cancer (Fig. 4A,C). These enrichment results validated the GSEA results and were visualized with corresponding DEGs using string diagrams to display the relationship between DEGs and ACLF pathogenesis (Fig. 4B,D).

**Figure 5:**
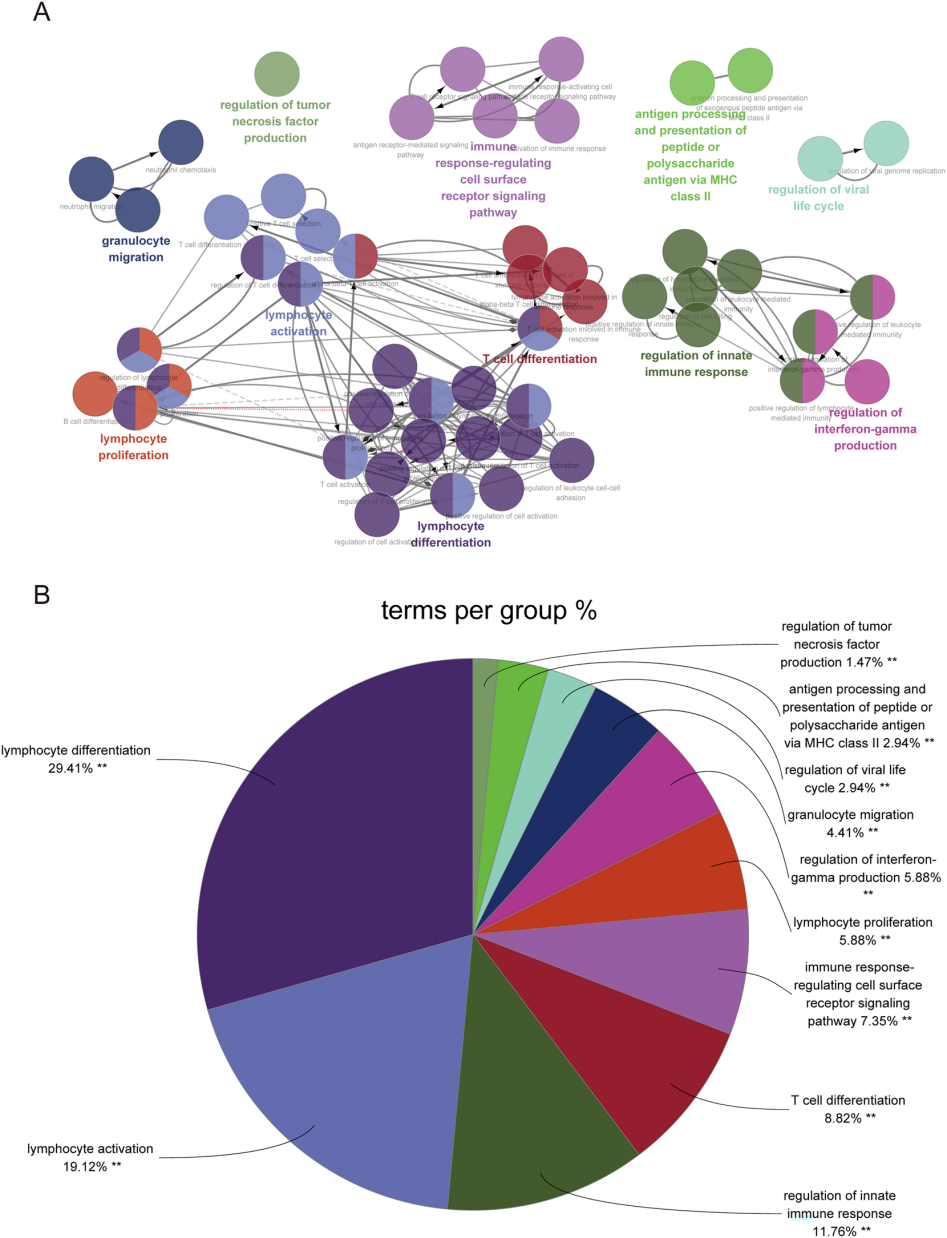
Enrichment analysis of DEGs biological processes (BPs) in GSE142255 was performed using ClueGO. Then, (**A**) a BPs network was constructed. (**B**) The proportion of relevant genes in major BPs are presented in pie charts.

Moreover, we performed a GO-BP enrichment analysis of DEGs and constructed an interaction network using the ClueGo plug-in in Cytoscape (Fig. 5). The network revealed that the biological processes of ACLF mainly included the activation, differentiation, proliferation, and migration of immune cells.

### Identification of gene co-expression modules by WGCNA

**Figure 6:**
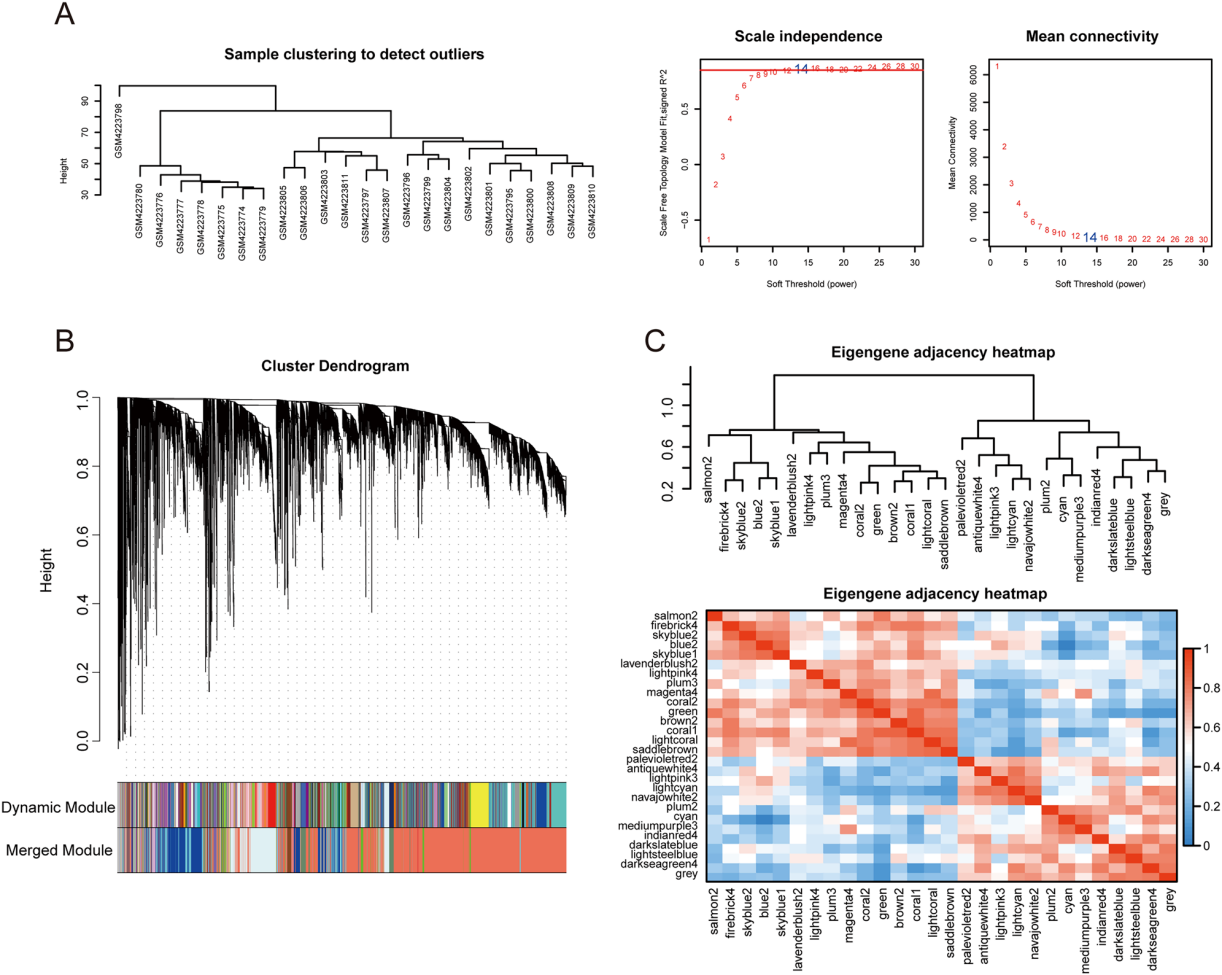
Gene co-expression modules associated with ACLF were identified by WGCNA. (**A**) The clustering analysis of ACLF patients and HS in GSE1422552 with a β = 14 as soft threshold (power) and scale-free topology fitting index (R^2^) = 0.9. (**B**) Twenty-eight gene co-expression modules were obtained. (**C**) The relationship between modules was visualized in a heat map.

The top 15,000 genes in GSE142255 were used to construct a co-expression network with a β power = 14 (Fig. 6A), resulting in 28 gene modules. Genes in the same module have similar expression profiles. Each module contained at least 20 genes with a merge cut height of 0.25. The clustering relationship between modules was represented in a heat map to show the correlation between different modules (Fig. 6B,C).

### Correlation analysis of immune infiltration and gene modules

**Figure 7:**
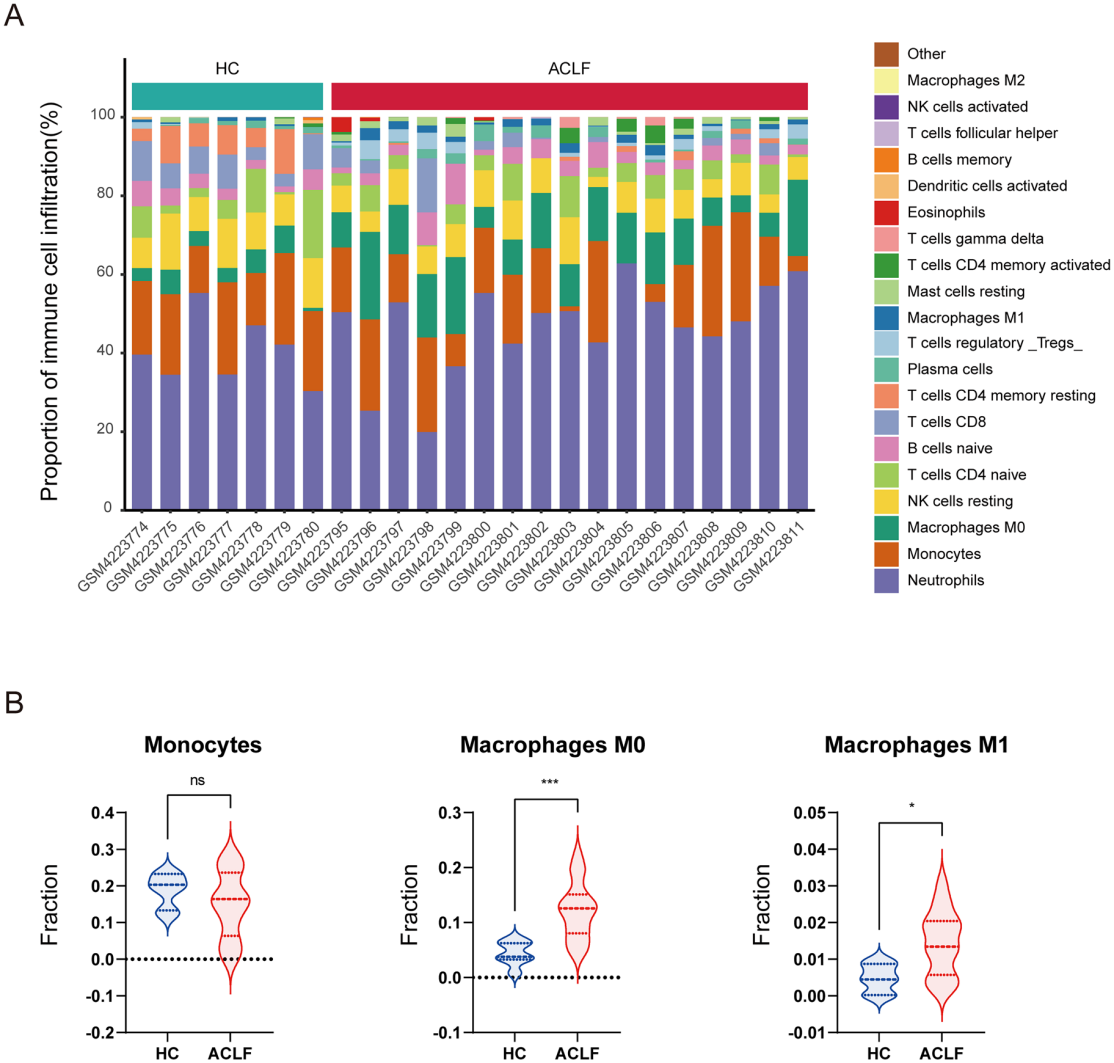
Immune cell composition in ACLF. (**A**) Proportions of 22 immune cells in ACLF patients and HS. (**B**) Differences in the proportion of M0 and M1 macrophages, and monocytes between ACLF patients and HS.

**Figure 8:**
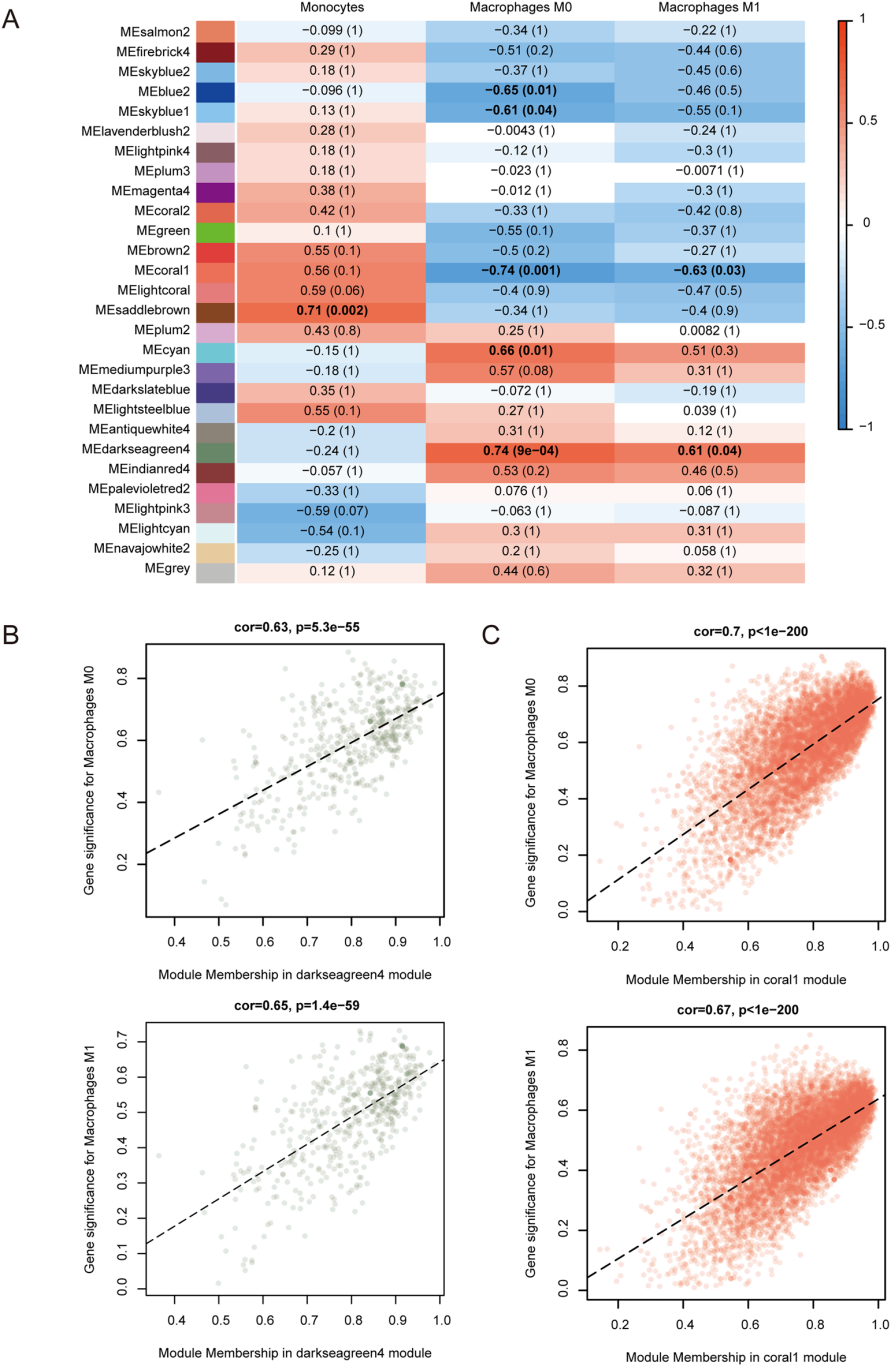
Correlation analysis between M0 and M1 macrophages, and key gene expression modules. (**A**) The heat map revealed that variations in M0 and M1 macrophages were associated with coral1 and darkseagreen4 modules, with corresponding correlation and *p*-value in each cell. (**B**) The scatter plot revealed that Gene significance (GS) and module membership (MM) are highly correlated in coral1 and darkseagreen4 modules.

As reported in GSE142255, ACLF patients are characterized by dysregulated blood immune cells, such as neutrophils, and several lymphocyte subsets. Herein, we used the CIBERSORT algorithm to investigate the correlation between immune infiltration and gene co-expression modules. The proportion of each immune cell type in each sample is shown in a stacked bar graph (Fig. 7A). The proportions of M0 and M1 macrophages increased, while the proportion of monocytes was not significantly altered, indicating significant macrophage differentiation and polarization in ACLF (Fig. 7B). Moreover, the coral1 module had significant negative correlations with M0 (*r* =− 0.63, *p* < 0.01) and M1 (*r* =− 0.65, *p* < 0.01) macrophages. On the other hand, the darkseagreen4 module had a significant positive correlation with M0 (*r* = 0.7, *p* < 0.01) and M1 (*r* = 0.67, *p* < 0.01) macrophages (Fig. 8).

### Construction and analysis of a PPI network of macrophage-related DEGs

**Figure 9:**
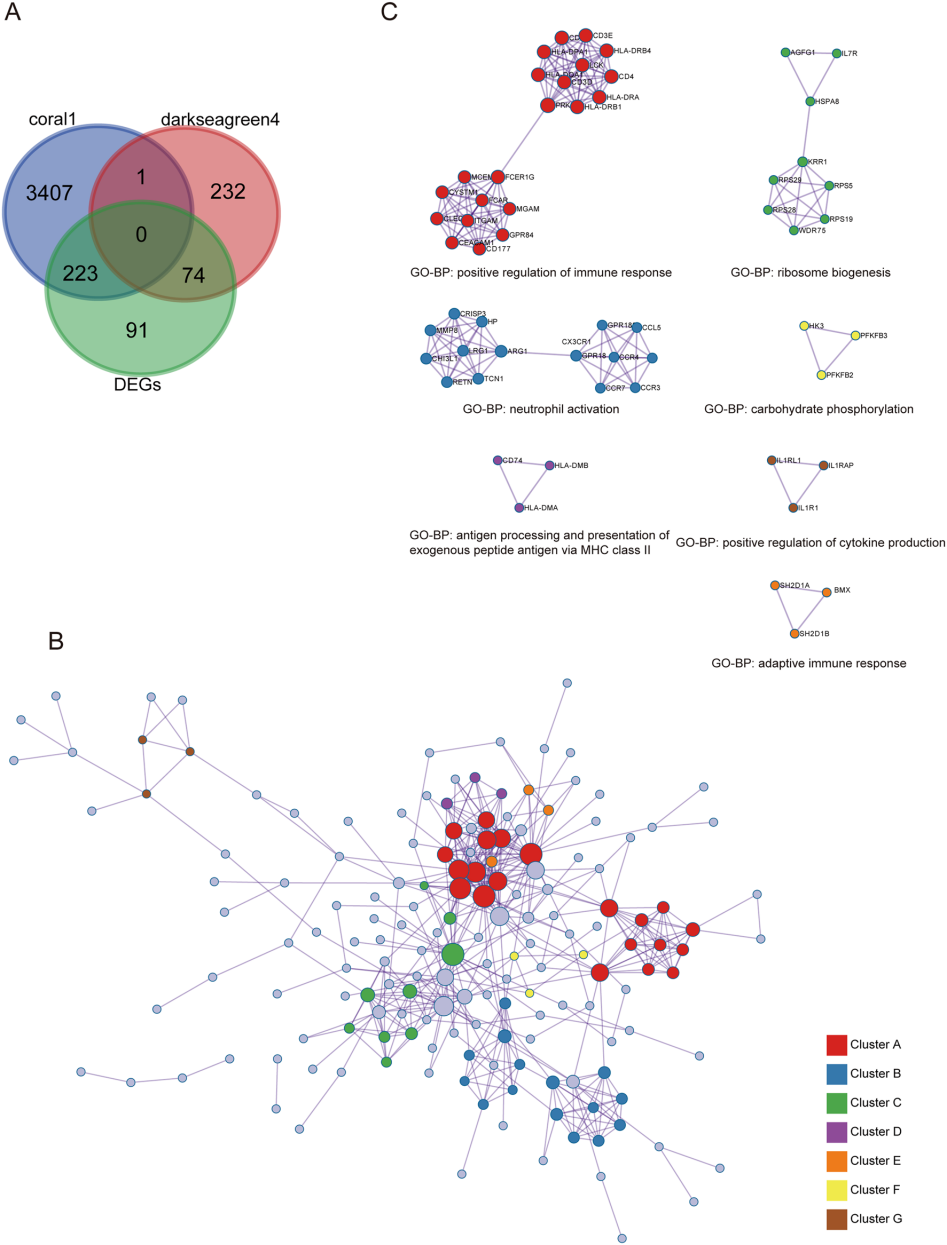
Acquisition of immune-related DEGs and construction of PPI networks. (**A**) The Venn diagram shows 297 DEGs overlapping with immune-related gene modules. (**B**) PPI network of 297 immune-related DEGs. (**C**) Seven clusters in the PPI network are described with BPs.

**Figure 10:**
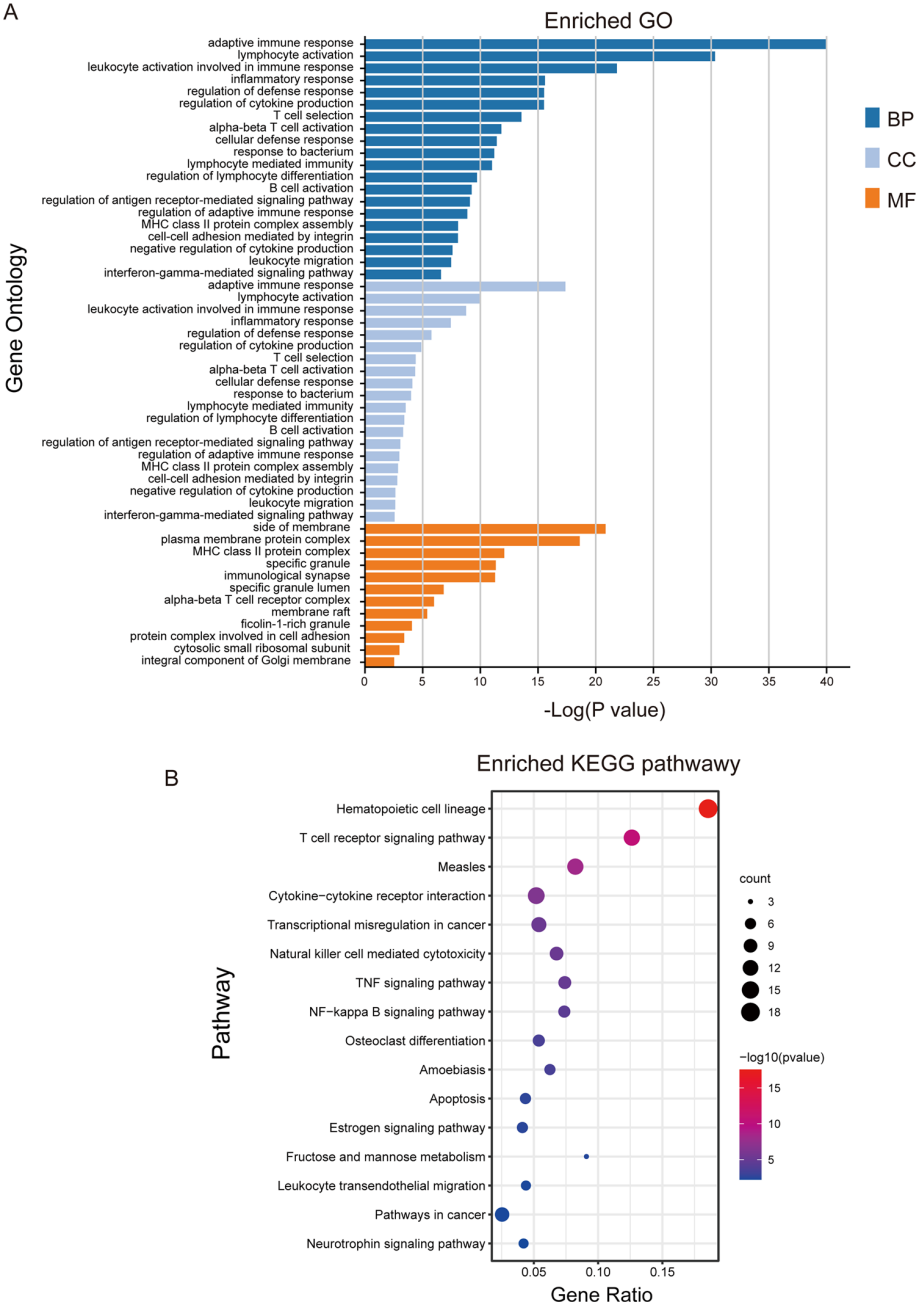
Enrichment analyses of the 297 DEGs. BP, MF, CC (**A**), and KEGG pathways (**B**) of the PPI were visualized.

To screen out macrophage-related DEGs, 297 genes were obtained from the intersection of the coral1 and darkseagreen4 modules and DEGs (Fig. 9A). Then, the PPI network was constructed, resulting in 168 nodes, 469 edges, and 7 gene clusters (Fig. 9B,C). The GO annotation and KEGG pathway analyses of the PPI network are shown in Fig. 10 and Supplementary Table S4.

### Identification of hub genes

**Figure 11:**
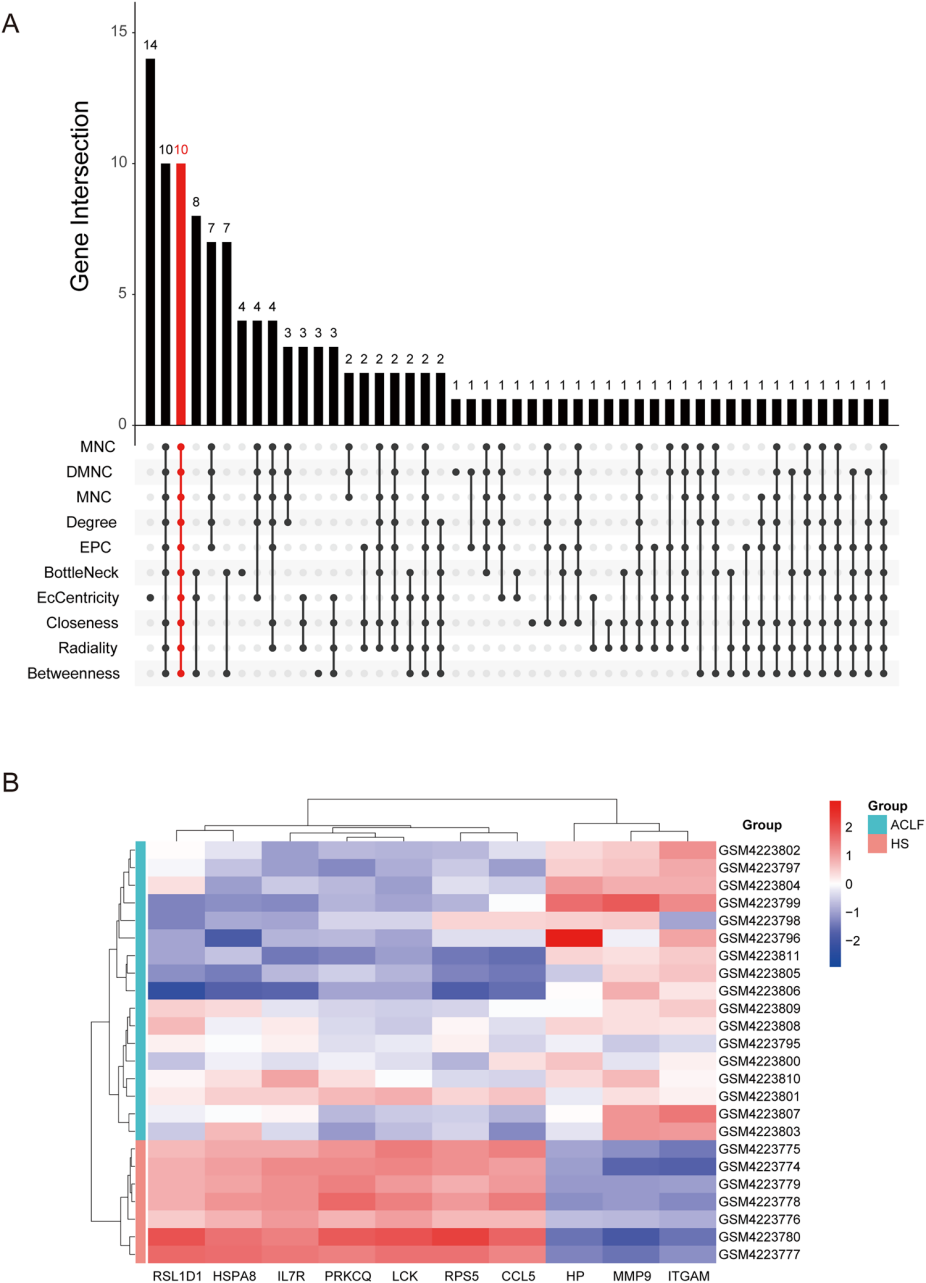
Identification of hub genes. (**A**) The hub genes were screened using 10 topological analysis methods. (**B**) The expression of 10 hub genes in GSE142255 is presented in a heatmap.

**Figure 12:**
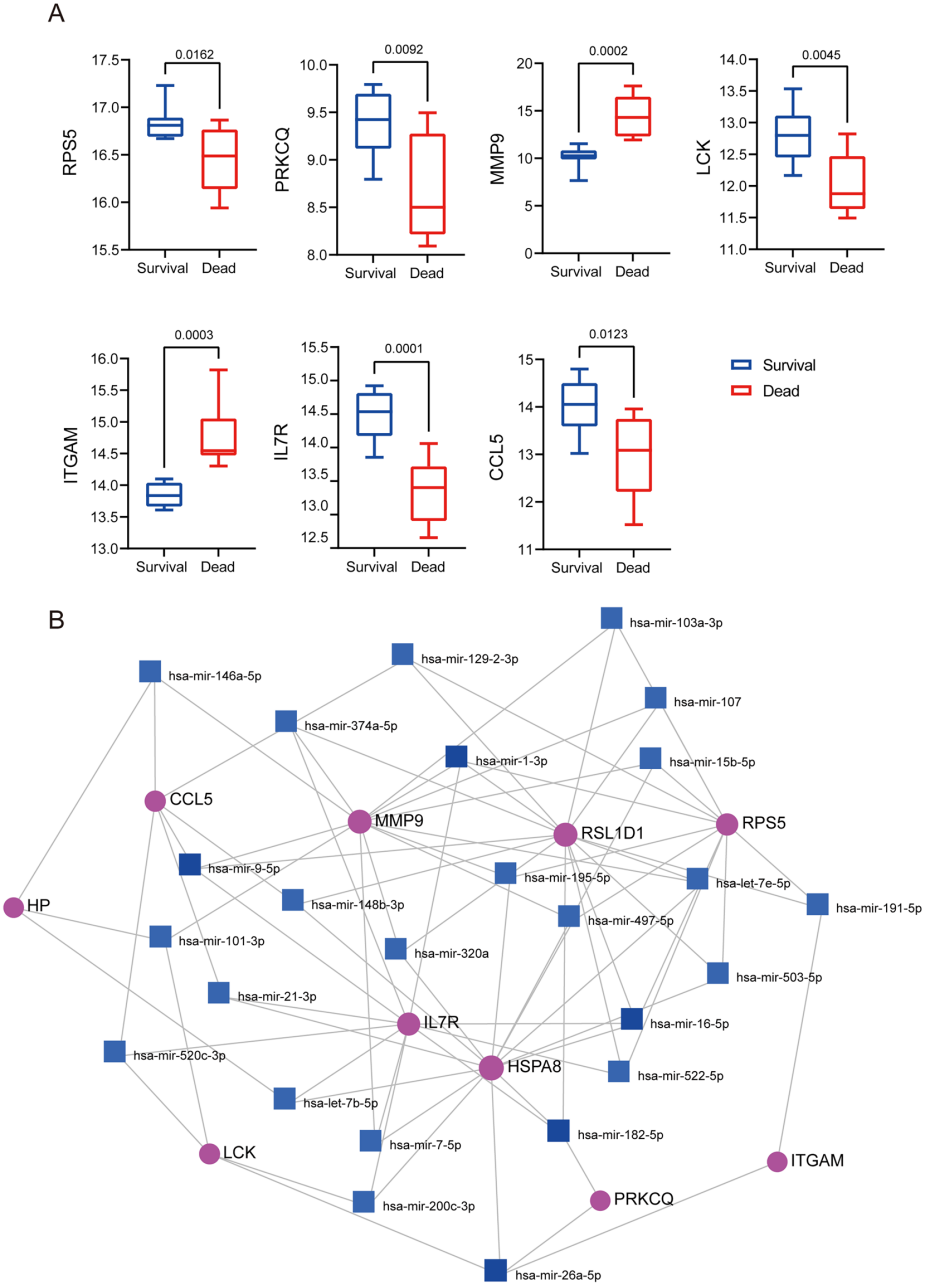
Validation of hub genes and prediction of miRNAs. (**A**) The expression of seven of these hub genes was statistically different in the GSE168048 samples. (**B**) miRNA–hub genes regulatory network. Purple circles represent hub genes, while blue squares represent miRNAs.

Based on the PPI network, we used ten topological analysis methods provided by CytoHubba to calculate and rank the parameters of each node. Then, ten hub genes were obtained: RSL1D1, RPS5, CCL5, HSPA8, PRKCQ, MMP9, ITGAM, LCK, IL7R, and HP (Fig. 11). Further, we explored the genes on GSE168048. We found that the expression of RPS5, PRKCQ, MMP9, LCK, ITGAM, IL7R, and CCL5 was statistically different between surviving and dead samples (Fig. 12A). The expression remained consistent in both datasets, suggesting that these genes are associated with the 28-day survival status of ACLF patients and might be critical in ACLF.

### Prediction of miRNAs related to hub genes

The ten hub genes were submitted to miRNet 2.0 for miRNA prediction and network construction. The miRNAs with degree values ≥ 3 are shown in Fig. 12B. The degree values of Has-mir-1-3p, has-mir-9-5p, has-mir-16-5p, has-mir-182-5p, and has-mir-26a-5p were equal to 4. Hence, they might be crucial miRNAs during ACLF development.

### Experimental verification in vivo

**Figure 13:**
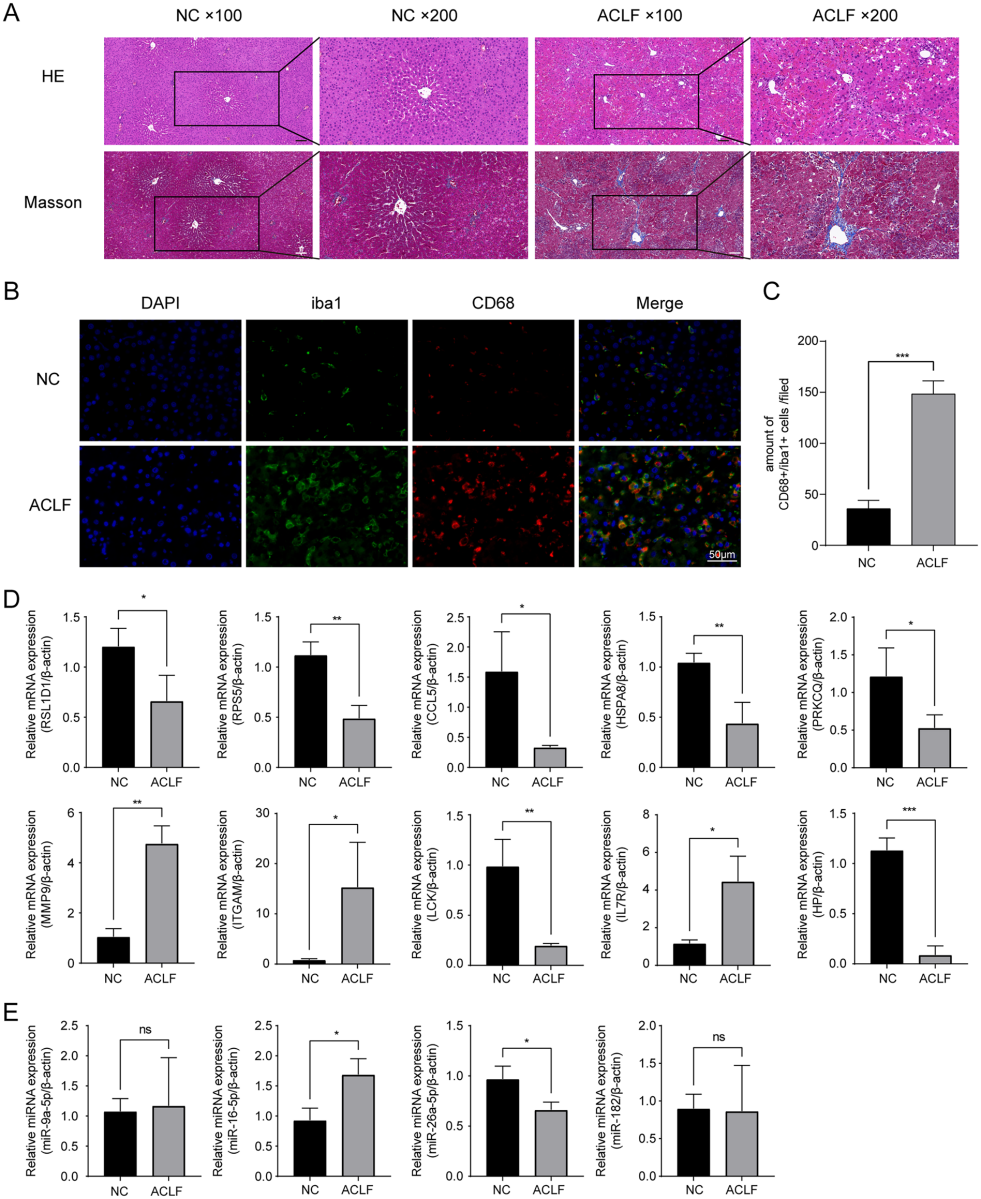
Experimental validation by establishing an ACLF rat model. (**A**) Pathological damage in rat liver observed by HE and Masson staining (Scale bars: 100 μm). (**B**) Liver sections were co-stained with iba1 (macrophages marker) and CD68 (M1 marker) (Scale bars: 50 μm, × 400). (**C**) Quantitative analysis of CD68 + /iba1 + macrophages in each field. The expression levels of hub gene (**D**) as well as miRNA (**E**) in ACLF rats were measured with qRT-PCR. *p < 0.05, **p < 0.01 and ***p < 0.001.

We assessed the pathological damage in ACLF model rats by HE, masson staining. The liver lobules of the rats in NC had a regular structure, and there were no necrotic cells, without obvious inflammatory cell infiltration or fibrous hyperplasia. However, the hepatocytes in the ACLF group were disorganized, with large and sub-large pieces of severe necrosis. A large infiltration of inflammatory cells is seen in the visual field, as well as marked hepatic sinusoidal haemorrhage. The hilar region is bridged by proliferating collagen fibres and pseudobullets are formed (Fig. 13A). Furthermore, liver sections were double-stained for iba1 (macrophages marker) and CD68 (M1 marker) by immunofluorescence^16,17^. A significant increase in iba1 and CD68 positive staining cells was detected in the livers of ACLF rats, suggesting that ACLF macrophages were recruited and induced to M1 polarization (Fig. 13B,C). Consistent with the results predicted by CIBERSORT, a significant macrophage infiltration occurred in ACLF rats. Subsequently, we used qRT-PCR to examine the expression levels of hub genes and miRNAs in ACLF rats compared to controls. The results showed a significant decrease in the mRNA expression of RSL1D1, RPS5, CCL5, HSPA8, PRKCQ, LCK, and HP (*p* < 0.05, *p* < 0.01, *p* < 0.001), while MMP9, ITGAM, and IL7R were significantly increased (*p* < 0.05, *p* < 0.01) (Fig. 13D). Then, to validate our predicted miRNAs, the miRNAs homologous to humans were matched using the miRBase database (https://mirbase.org/) and analyzed via qRT-PCR. The expression of mir-16-5p increased (*p* < 0.05) and mir-26a-5p decreased in ACLF rats (*p* < 0.05). In contrast, the expression of mir-182 and mir-9a-5p did not differ from controls (Fig. 13E).

## Discussion

ACLF is a systemic inflammatory disease accompanied by immune dysfunction and disturbances in energy metabolism. ACLF has a high short-term mortality, which increases with the incidence of failing organs. Although many studies regarding ACLF have been performed, its underlying mechanisms remain to be fully explored. Meanwhile, it has been demonstrated that a strong immune response is a key mechanism of ACLF^18,19^. Therefore, we explored the intrinsic mechanisms of immune cell infiltration in ACLF using an effective informational biology approach.

Herein, we identified macrophage-associated co-expressed gene modules in ACLF for the first time using a combination of WGCNA and CIBERSORT. We identified immune-related key genes and provided new pathways for future studies on effective targets for ACLF treatments. After bioinformatics and qRT-PCR experiments, 10 immune-related hub genes were identified and mir-16-5p and mir-26a-5p were validated. Altogether, these results might provide new strategies for understanding the pathogenesis of ACLF and developing targeted therapeutic molecules.

In the present study, we evaluated potential pathways and biological processes of ACLF using enrichment analyses. GSEA is characterized by the analysis of collections of genes rather than individual genes, which helps to avoid the inability to reproduce individual high-scoring genes due to poor annotation. In the GSE142255 dataset, the GSEA indicated that immune response, inflammatory pathways, and metabolic pathways were mainly involved in ACLF. Then, we found that the downregulated DEGs were mainly engaged in immune response and inflammatory reaction, while upregulated ones regulated biosynthetic and substance metabolism pathways. These results reflected two major biological processes that co-occurred during the progression of ACLF regulated by different genes: imbalance of immune-inflammatory response and energy metabolism. According to the BP analysis, immune cell activation, differentiation, proliferation, and migration were the major biological processes in ACLF, leading to an expanding inflammatory response. Recently, it was reported that excessive activation of the immune response not only causes a systemic inflammatory response, which subsequently mediates immune-related tissue damage, but also leads to high energy demand. Consequently, the immune system competes with peripheral organs for energy, triggering an immune-related energy crisis in the organism and increasing the risk of organ failure^18,20,21^. Overall, it was suggested that the hyperimmune response and dysfunctional energy metabolism in ACLF are biologically coupled processes, largely influencing ACLF progress.

CIBERSORT is a widely used deconvolution machine algorithm for estimating the composition of immune cells. It shows superior performance in the identification and fine delineation of immune cells when processing highly noisy mixture data^8,22^. Here, the CIBERSORT results showed that the population of M0 and M1 macrophages was significantly increased in ACLF patients compared to healthy subjects. We also labeled markers on the surface of macrophages by immunofluorescence and validated their increase in M1 macrophages in the liver of ACLF rats. Macrophages can be polarized into M1 or M2 phenotypes. M1 macrophages can release significant influxes of inflammatory factors and induce cytokine storms with pro-inflammatory effects. On the other hand, M2 macrophages secrete tissue repair factors and exhibit anti-inflammatory and reparative properties^23,24^. Kupffer cells, a type of macrophage that resides in the hepatic sinusoids, mainly perform innate immune and inflammatory responses^25^. To search for highly related gene modules, WGCNA identifies similar gene clusters and gene modules by hierarchical clustering. WGCNA also supports the analysis of correlations between gene modules and phenotypic traits^26,27^. To identify gene clusters associated with macrophages, we performed WGCNA and identified gene modules closely related to M1 macrophage polarization, including the coral1 (containing 3631 genes) and darkseagreen4 (containing 307 genes) modules. Based on the WGCNA’s gene modules, we screened immune-related DEGs and constructed a PPI network to find ACLF immune-related hub genes.

Ten hub genes were screened using CytoHubba: RSL1D1, RPS5, CCL5, HSPA8, PRKCQ, MMP9, ITGAM, LCK, IL7R, and HP (Table 3). The differential expression of hub genes was further confirmed by qRT-PCR in ACLF rats. Overall, MMP9, ITGAM, and IL7R were highly expressed during ACLF. Furthermore, ACLF has high 28-day mortality that is closely related to the degree of organ failure in patients. Hence, we used the GSE168048 microarray containing gene expression data of ACLF patients who survived or died at 28 days for further investigation. We verified that the expression of RPS5, PRKCQ, MMP9, LCK, ITGAM, IL7R, and CCL5 differed between surviving and deceased patients, suggesting that these genes might be closely related to ACLF progression and could be used to predict ACLF survival status at 28 days. Notably, downregulated genes were mostly involved in the promotion of immune response, while the upregulated gene, MMP9, was associated with hepatocyte necrosis. These results suggested that the coexistence of immune paralysis and cell necrosis is a potential ACLF mechanism leading to poor prognosis.

**Table 3:** Description of hub genes.

Gene symbol	Description	Function	Log FC	Adj.P value
RSL1D1	Ribosomal L1 Domain Containing 1	Promotes cell growth, participates in cell proliferation and senescence, and inhibits PTEN translation^38,39^	− 1.06034482	0.00106
RPS5	Ribosomal Protein S5	Interacts with the HCV internal ribosome entry site (IRES) sequence and mediates the intrinsic translation machinery^40^	− 1.40499361	0.0000138
CCL5	C–C Motif Chemokine Ligand 5	CCL5 induces activation of MAPK and NF-κB pathways to directly induce M1 polarization and prevent M2 polarization in Kupffer cells^41^.	− 1.87593731	0.0000241
HSPA8	Heat Shock Protein Family A (Hsp70) Member 8	Acts as a receptor for LPS and participates in LPS-mediated immune responses^42^	− 1.32267171	0.000272
PRKCQ	Protein Kinase C Theta	Control the T-cell proliferation and differentiation^43^, promotes polarization of M1 macrophages, and enhances antimicrobial immunity^44^	− 1.21956335	0.00000067
MMP9	Matrix Metallopeptidase 9	LPS stimulation leads to the release of MMP9^45^, which in turn damages the hepatic sinusoids and aggravates intrahepatic hemorrhage and necrosis^46^.	1.93977841	0.00000258
ITGAM	Integrin Subunit Alpha M	Involved in adhesion interactions of monocytes and macrophages^47,48^, initiating LC3-related phagocytosis of monocytes^49^	1.03325032	0.00000639
LCK	LCK Proto-Oncogene, Src Family Tyrosine Kinase	Regulates T-cell development and activation and participates in T-cell antigen receptor-related signaling^50,51^	− 1.08220517	0.000000115
IL7R	Interleukin 7 Receptor	Participates in the development of T cells, which form the defense barrier of the lymphatic system^52^	− 1.7536909	0.0000458
HP	Haptoglobin	Formed during the acute phase of inflammation and binds free hemoglobin to form a complex^53^	1.88975205	0.000564

Moreover, miRNAs are potential targets in numerous diseases and control various biological processes. As short-chain RNAs with a coding length of only about 22 nucleotides, miRNAs cannot directly be translated into proteins, but rather regulate protein synthesis by disrupting the stability of target mRNAs and inhibiting their translation through complementary pairing^28^. Studies have explored the relationship between miRNAs and diseases and proposed the use of miRNAs as a biomarker for disease diagnosis and prognosis as well as a small molecule drug target^29^. Considering the time and cost of experimental studies, we adopted a database approach combined with experimental validation to study miRNAs that were significantly altered in ACLF. The miRNet 2.0 integrates data from 15 prediction databases and provides visual analytics to enable a more comprehensive and convenient evaluation of the interactions between miRNAs, mRNAs, lncRNAs, and transcription factors^15^. Herein, we used miRNet 2.0 to construct a miRNA-hub genes network to explore potential miRNAs related to ACLF. During the validation, two miRNAs were significantly altered in ACLF rats: mir-16-5p presented increased expression and mir-26a-5p showed decreased expression. M1 macrophages can transfer mir-16-5p to gastric cancer (GC) cells by secreting exosomes and triggering a T-cell immune response to suppress tumor formation by decreasing the expression of PD-L1^30^. It has been demonstrated that mir-26a-5p decreases with ACLF progression and is associated with worsening liver function and increasing liver disease severity^31^. However, further studies are needed to validate the potential association between miRNA regulatory networks and ACLF.

Predicting potential disease-associated miRNAs is very meaningful and challenging. Thus, researchers have developed several computational methods and models to perform those predictions. These models can be classified into four categories: score functions, complex network algorithms, machine learning, and multiple biological information^29^. For example, Chen et al.^32^ proposed an inductive matrix filling model (IMCMDA) for miRNA-disease association prediction. By integrating miRNA and disease similarity information into the matrix-populated objective function, a low-dimensional representation matrix of miRNAs and diseases was obtained, which was finally combined into a miRNA-disease association score matrix. Chen et al.^33^ improved the HGIMDA model and further provided the MDHGI model. This model first decomposes the miRNA-disease association matrix to remove data noise, then uses the topological information implied to make predictions through heterogeneous graph inference. It combines machine learning with network analysis methods to make effective predictions for new disease-miRNA associations. Further, Chen et al. proposed an Ensemble of Decision Tree-based MiRNA-Disease Association prediction (EDTMDA) model^34^ based on the construction of multiple decision trees by randomly selecting negative samples, miRNA features, and disease features, and by dimensionality reduction of the features. The mean of the predicted values from these decision trees is used as the miRNA-disease association score. This model incorporates feature dimensionality reduction into integrated learning to remove noise and redundant information in the learning process and reduce the computational complexity of the model with higher prediction accuracy. Moreover, Liu et al.^35^ proposed a DFELMDA-based deep forest integrated learning approach to infer miRNA-disease correlations. This model trains a random forest by constructing two auto-encoders based on miRNAs and diseases, extracting low-dimensional feature representation, and finally predicting potential miRNA-disease associations through the random forest. This model combines feature and deep forest-integrated learning models to enhance the prediction accuracy. Bioinformatics-based prediction methods are constantly evolving. Nevertheless, different models have almost different predictive performance for the same datasets. Hence, it is not only necessary to collect large-scale experimental data but also consider other algorithms to improve predictive performance for specific diseases.

Besides the methods covered in this study, the multi-field predictive research of bioinformatics offers a unique perspective on the exploration of diagnostic and therapeutic tools for diseases, not only for ACLF. Currently, with the development of genome-wide technologies, there is an increasing need to explore models that detail the exact mechanisms in which genes and proteins interact to form complex living systems. A gene regulatory network (GRN) is a network of interactions between gene molecules. An improved Markov blanket discovery algorithm based on IMBDANET has been proposed and can effectively distinguish between direct and indirect regulatory genes from GN and reduce the false-positive rate in the network inference process^36^. Additionally, RWRNET is an algorithm of Random Walk with Restart (RWR) modified by restart probability, initial probability vector, and roaming network applied to GRN that continuously maps the global topology of the network and estimates the affinity between nodes in the network through circular iterations until all nodes are traversed^37^. In contrast, IMBDANET uses a Markov blanket discovery algorithm for network topology analysis and processing, identifying direct and indirect regulatory genes while solving the problem of isolated nodes. On the other hand, RWRNET focuses on global network topology information but it cannot handle isolated nodes. Finally, the integration of different methods can be more beneficial for the prediction of gene regulatory relationships.

Here, we combined WGCNA and CIBERSORT algorithms and employed GSEA, KEGG, and GO enrichment analyses to explore immune-related hub genes and potential biological mechanisms in ACLF. The hub genes and miRNAs involved in ACLF regulation were also further validated. Since there are few studies regarding ACLF mechanisms, adopting bioinformatics analyses provided valid information and guidance for our research. However, our current study also has some limitations. First, we used an animal model rather than samples from humans to validate the ACLF immune-related hub genes, and the results from animal studies should be treated with caution. Furthermore, although these hub genes and miRNAs were altered and might be involved in the development of ACLF, whether these genes can be new therapeutic targets for ACLF still needs to be explored. Therefore, further experiments are required to validate our findings and explore potential ACLF mechanisms.

## Conclusion

In summary, we used different bioinformatics approaches to uncover potential ACLF molecular mechanisms. The biological processes and pathways that govern immune activation provided a meaningful insight for studying ACLF pathogenesis. Immune-related hub genes and important miRNAs that might be involved in critical ACLF functions were also identified. Finally, further studies are needed to identify the molecular mechanisms of these key genes, which might also contribute to the understanding of ACLF.

## Supplementary Information

Supplementary Information 1.

Supplementary Information 2.

Supplementary Information 3.

Supplementary Information 4.

## Data Availability

The datasets (GSE142255 and GSE168048) analysed during the current study are available in the Gene Expression Omnibus (GEO) repository(http://www.ncbi.nlm.nih.gov/geo/).

## Abbreviations

ACLF: Acute-on-chronic liver failure
PPI: Protein–protein interaction
HS: Healthy subjects
DEGs: Differentially expressed genes
WGCNA: Weighted correlation network analysis
GSEA: Gene set enrichment analysis
GO: Gene ontology
BP: Biological processes
MF: Molecular functions
CC: Cellular components
GS: Gene significance
MM: Module membership
EPC: Edge percolated component
MNC: Maximum neighborhood component
DMNC: Density of maximum neighborhood component
MCC: Maximal clique centrality
BN: Bottleneck
OXPHOS: Oxidative phosphorylation
